# Comparative analysis of genetic architectures for nine developmental traits of rye

**DOI:** 10.1007/s13353-017-0396-3

**Published:** 2017-05-09

**Authors:** Piotr Masojć, P. Milczarski, P. Kruszona

**Affiliations:** Department of Genetics, Plant Breeding and Biotechnology, West Pomeranian University of Technology, Słowackiego 17, 71-434 Szczecin, Poland

**Keywords:** *Secale cereale* L., DArT, High-density map, Quantitative traits, Divergent selection, Classes of QTL

## Abstract

Genetic architectures of plant height, stem thickness, spike length, awn length, heading date, thousand-kernel weight, kernel length, leaf area and chlorophyll content were aligned on the DArT-based high-density map of the 541 × Ot1–3 RILs population of rye using the genes interaction assorting by divergent selection (GIABDS) method. Complex sets of QTL for particular traits contained 1–5 loci of the epistatic D class and 10–28 loci of the hypostatic, mostly R and E classes controlling traits variation through D–E or D–R types of two-loci interactions. QTL were distributed on each of the seven rye chromosomes in unique positions or as a coinciding loci for 2–8 traits. Detection of considerable numbers of the reversed (D′, E′ and R′) classes of QTL might be attributed to the transgression effects observed for most of the studied traits. First examples of E* and F QTL classes, defined in the model, are reported for awn length, leaf area, thousand-kernel weight and kernel length. The results of this study extend experimental data to 11 quantitative traits (together with pre-harvest sprouting and alpha-amylase activity) for which genetic architectures fit the model of mechanism underlying alleles distribution within tails of bi-parental populations. They are also a valuable starting point for map-based search of genes underlying detected QTL and for planning advanced marker-assisted multi-trait breeding strategies.

**Electronic supplementary material:**

The online version of this article (doi:10.1007/s13353-017-0396-3) contains supplementary material, which is available to authorized users.

## Introduction

Bidirectional selective genotyping (BSG), a method based on analysis of the most diversified phenotypes from the opposite population tails, is an effective tool for QTL detection in plant and animal species (Foolad et al. 2003; Gallais et al. 2007; Navabi et al. 2009; Venuprasad et al. 2009; Yang et al. 2010; Eskandari et al. 2013; Myśków and Stojałowski 2016). For bi-parental populations, beside differences in alleles frequencies within the opposite tails, segregation distortions from the Mendelian ratios can be used as criteria for QTL detection. This new type of genetic analysis allowed to discern three classes of QTL, showing different responses to divergent selection (Masojć et al. 2009, 2011). Loci of class D (directional) exhibit distorted allelic segregations in lower and upper tails, which means that the detected polymorphism is significant for both directions of divergent selection. Loci of class R respond only to selection in one direction, positive in respect to breeding value, showing segregation distortion in the population tail representing the best phenotypes and a 1:1 segregation ratio in the opposite tail accumulating undesirable phenotypes. Polymorphism in QTL of class E is neutral for selection in the positive direction but reveals significant segregation distortion within the population tail with undesirable phenotypes. Genetic model of mechanism generating classes of QTL identifies two-loci interactions as a basic factor affecting quantitative traits variation (Masojć et al. 2016). Predominant interactions are of the D–R type, controlling change of trait value in the positive direction and D–E type, affecting trait variation in the negative direction, where the QTL of class D is epistatic. The premium role of the class D loci is well documented for pre-harvest sprouting (PHS) in rye as it is represented by such key genes as *ScGA3ox* and *ScGAMYB* known to affect the expression of many genes involved in grain germination (Masojć et al. 2016).

A method of identifying classes of QTL and their possible types of interaction based on the new genetic model (Masojć et al. 2016) is a step forward in respect to the classic BSG method and is referred to as the genes interaction assorting by divergent selection (GIABDS) method. Until now, GIABDS proved to be efficient in characterising genetic architectures of pre-harvest sprouting and alpha-amylase activity in rye (Masojć et al. 2009, 2011). In both studies, QTL distributed on each of the rye chromosomes represented the D, R or E classes of loci and the same loci belonged to equal or alternative classes for different traits and intercrosses studied.

The aim of this paper is to characterise and compare the genetic architectures of nine developmental traits of rye using the GIABDS method and a high-density DArT-based genetic map developed on the 541 × Ot1–3 population. This study also aimed at further verification of the genetic model developed for the analysis of population tails in bi-parental populations.

## Materials and methods

The bi-parental 541 × Ot1–3 mapping population of rye consisting of 144 RILs and representing advanced (F_>11_) generation of inbreeding was used as the plant material (Milczarski et al. 2011). Parental lines of this cross differ in respect to many phenotypic traits, which was observed through years of propagations. Line 541 derived from the KaH9 × [(MS69-8-1 × Smolickie)F_2_ × KaH9] cross is tall (120–140 cm), has long spikes (11–12 cm), long awn (4–5 cm), high thousand-kernel weight (21–23 g) and large, bending leaves of light green colour. This line is extremely susceptible to sprouting and develops high levels of alpha-amylase activity in mature grain. Line Ot1–3 was selected from the Swedish cultivar Othello and represents low height (70–80 cm), short spike (5–7 cm) with reduced awn (1–2 cm), grain of low thousand-kernel weight (13–15 g), and small upright leaves with dark green colour. Ot1–3 is highly resistant to pre-harvest sprouting and develops low alpha-amylase activity during ripening. Both lines are medium late, with heading dates around the first and second weeks of June in the weather conditions of Szczecin, Poland. Wide genetic distance between 541 and Ot1–3 lines explaining their substantial differentiation in respect to numerous traits was confirmed using different types of molecular markers and a large collection of rye inbred lines (Myśków et al. 2001, 2010).

RILs of the mapping population and parental lines were cultivated in rows of 1 m length with 20 cm inter-space on the experimental field of West Pomeranian University of Technology, Szczecin, Poland in 2015 and 2016. Five plants from each row were phenotyped in consecutive years and the two subgroups of lines representing the lowest and highest values for each trait were selected. Measurements of heading date (day of emergence of at least three spikes per row), height of mature plants (distance from the ground level to the top of spike), stem thickness (third internode from the ground in mature plants), area of the third leaf from the top and chlorophyll content (assayed a few days before pollen shedding using Konica Minolta Chlorophyll Meter SPAD-502, Konica Minolta, Inc., Japan) were performed in the field. Measurements of the spike length, awn length (from one-third of spike from the top), thousand-kernel weight and kernels length were performed after harvest in the laboratory.

The two selected subgroups of RILs representing lower and upper population tails for each trait were compared in respect to genotypes at 1465 molecular marker loci arranged in a DArT-based high-density genetic map of rye developed earlier using 144 RILs of the 541 × Ot1–3 mapping population (Milczarski et al. 2011). It was assumed that allelic segregation consistent with the 1:1 Mendelian ratio within each of the two selected subgroups would define markers not related to the trait, whereas significant distortion from this ratio in at least one of the two subgroups would reflect a marker–trait relationship. Segregation distortion in at least three consecutive marker loci ordered on the map was a basic condition for declaring detection of QTL. A given QTL was thus defined by the block of linked markers showing the same pattern of distorted segregation. Classes of QTL were discerned on the basis of segregation patterns, according to the GIABDS method (Masojć et al. 2016). Over-representation of an A (541 parental line) allele in one subgroup and prevalence of the B (Ot1–3 parental line) allele in the second subgroup defined the D class of QTL. Class R was recognised after detecting significant segregation distortion in a subgroup with desirable phenotypes and accordance with the 1:1 allelic segregation ratio within the opposite subgroup. QTL was classified as E when one of the alleles was prevalent among RILs with undesirable phenotypes, while both alleles were found with similar frequencies among lines of the opposite subgroup. When an allele originating from a parental line was over-represented among the subgroup of RILs with the opposite phenotype, the reversed classes of QTL were declared (D′, R′, E′). Segregation distortions were checked within each subgroup separately by the χ^2^ test at a *p* ≤ 0.05 significance level.

The interpretation of results is based on the genetic model showing that QTL classes detected using the GIABDS method reflect D–E or D–R types of two-loci interactions, affecting the trait value in the negative or positive directions, respectively (Masojć et al. 2016).

## Results

The variation range of the mapping population exceeded that of parental lines for most of the studied traits, which can be determined by comparing the lowest values in the lower tail and the highest values in the upper tail (Table 1). It was especially evident for heading date, since spikes of the earliest lines emerged on 28–30th May and those of the latest lines on 15–18th, June while both parental lines were medium late (7–14th June). Wide variation ranges were found for plant height (50.0–154.8 cm), spike length (5.2–13.0 cm), thousand-kernel weight (9.1–24.0 g), leaf size (4.8–33.9 cm^2^) and chlorophyll content (17.5–56.1 SPAD units). These data suggest the existence of transgression among the studied progeny of RILs. Subgroups of 12–20 RILs representing the lower or upper tails of the 541 × Ot1–3 mapping population differed substantially in respect to each of the nine studied quantitative traits (Table 1). The lowest values of the variation range in the upper tail were always much higher than the highest values found in the lower tail and 1.3–4.8-fold differences were found between respective means. Plants from the upper tail were 47–65 cm higher than those from the lower tail. Divergent selection for spike length gave differences of 3.8–4.8 cm between the opposite groups. Substantial differences were also observed for awn length (2.2–3.0 mm), heading date (22 days), thousand-kernel weight (7.2–11.5 g), leaf size (13.2–21.3 cm^2^) and chlorophyll content (24.6 SPAD units). The latter difference was high enough to visually discern lines with dark green leaves from lines with light green leaves and, in this way, the results from year 2015 were verified in 2016. Strong differentiation of selected subgroups in respect to trait values is a basic condition for using the GIABDS method for QTL detection. The data presented in Table 1 prove that plant materials selected for this study fulfil this condition. Another important precondition for using the GIABDS method is the sufficient number of lines in each subgroup. Earlier results (Masojć et al. 2009, 2011) demonstrated that the number of RILs for detecting significant segregation distortions should not be less than 12. This condition is fulfilled for each selected subgroup, which allows to perform reliable genetic analysis.

**Table 1:** Phenotypic characterisation of recombinant inbred lines representing lower and upper tails of the 541 × Ot1–3 mapping population, in respect to rye developmental traits

Trait	Year	Lower tail				Upper tail			
		No. of RILs	Variation range		Mean	No. of RILs	Variation range		Mean
Plant height (cm)	2016	16	50.0	85.8	71.6	15	119.2	154.8	136.3
	2015	16	62.8	90.0	80.1	15	111.5	156.7	126.9
Stem thickness (mm)	2016	12	3.1	3.9	3.6	13	5.0	6.2	5.5
	2015	12	3.5	3.9	3.7	13	4.8	7.4	5.6
Spike length (cm)	2016	13	5.2	6.4	5.9	20	9.1	13.0	10.7
	2015	13	6.0	6.9	6.4	20	8.9	13.4	10.2
Awn length (cm)	2016	13	1.1	1.9	1.5	18	4.2	4.7	4.5
	2015	13	1.2	2.3	1.8	18	3.6	5.3	4.1
Heading date (day, month)	2016	15	18.05	20.05	19.05	13	5.06	15.06	10.06
	2015	15	20.05	22.05	21.05	13	6.06	16.06	12.06
Thousand-kernel weight (g)	2016	15	10.0	14.2	12.9	16	18.4	25.3	20.1
	2015	15	9.1	13.7	11.4	16	21.0	24.0	22.9
Kernel length (mm)	2016	13	6.2	7.3	6.9	15	8.1	10.8	8.8
	2015	13	5.9	7.0	6.5	15	8.2	10.0	8.8
Leaf size (cm^2^)	2016	13	4.8	7.2	5.6	17	20.3	33.9	26.9
	2015	13	6.1	9.9	8.2	17	18.3	25.5	21.5
Chlorophyll content (SPAD units)	2015	13	17.5	31.7	27.0	16	41.7	56.1	51.6

Finding common RILs with extreme values for two traits may suggest partial overlapping of genetic architectures. Examination of subgroups selected for different traits showed that they contain mostly distinct RILs (Table 2). Usually, 0–3 lines were common for the compared subgroups, which gave 0.00–0.09 common/unique RILs ratios, reflecting rather independent genetic control of the two traits. However, for certain pairs of traits, this ratio was much higher (0.17–0.30), indicating partial similarity of their genetic backgrounds. Genetic relationships can be suggested for: spike length and plant height, spike length and stem thickness, spike length and thousand-kernel weight, chlorophyll content and leaf area, long spike and large leaf size, long spike and long awn, long awn and high plants, long awn and large leaves, and low chlorophyll content and long spike.

**Table 2:** Relationship between quantitative traits suggested by increased ratio (in **bold**) of lines representing both compared phenotypes per number of lines unique for each of the compared phenotypes grouped through divergent selection within the 541 × Ot1–3 mapping population. The l and h subscripts indicate subgroups of lines with low or high phenotypic values, respectively

	PH_h_	PH_l_	ST_h_	ST_l_	TKW_h_	TKW_l_	LS_h_	LS_l_	SL_h_	SL_l_
SL_h_	**0.17**	0.09	**0.16**	0.04	**0.24**	0.00	**0.30**	0.06		
SL_l_	0.04	**0.17**	0.00	**0.25**	0.03	**0.21**	0.00	0.08		
AL_h_	**0.27**	0.03					**0.18**	0.07	**0.23**	0.00
AL_l_	0.04	0.00					0.00	0.04	0.06	0.08
CC_h_							0.00	**0.21**	0.09	0.07
CC_l_							**0.27**	0.00	**0.22**	0.00
ST_h_	**0.19**	0.03					0.11	**0.21**		
ST_l_	0.04	0.08					0.00	0.04		
KL_h_	0.13	0.06							**0.30**	0.05
KL_l_	0.04	**0.18**							0.07	**0.20**

Genotyping of each RIL with 1465 molecular markers, arranged on the high-density map of rye genome, revealed that alleles segregation in the majority of polymorphic loci was consistent with the 1:1 Mendelian ratio within particular subgroups of lines. Such a result revealing the lack of response to divergent selection is expected for loci not related to the studied trait. Statistically significant distortion from the 1:1 ratio of alleles segregation was found in population tails when the frequency of one allele exceeded at least three times the frequency of the second allele. A block of three or more linked marker loci with distorted segregation constituted individual QTL (Fig. S1). QTL on particular chromosomes were arranged from the distal part of the short arm to the distal part of the long arm by assigning them consecutive numbers. They are designated by trait symbol, chromosome and locus number, and a proximal dotted line showing their range. The last letter of the symbol informs about the QTL class distinguished on the basis of comparing segregation patterns in lower and upper tails according to the genetic model presented earlier (Masojć et al. 2016). D is the class representing the epistatic locus that shows segregation distortions in lower and upper tails with different alleles prevalent in each subgroup. Class R is hypostatic against D class locus and shows segregation distortion only in a group of extreme phenotypes resolved through positively directed selection, i.e. low height, long spike, thick straw, early heading date, high thousand-kernel weight, long grain, long awn, large leaf area and high chlorophyll content. Class E (hypostatic in respect to class D) represents loci where segregation distortion is found only in a group of extremes with not desirable phenotype, i.e. high plant, short spike, thin straw, late heading, low thousand-kernel weight, short awn and small leaves with low chlorophyll content. An additional (′) mark informs about the reversed allele effect in a progeny in respect to that expected on the basis of its origin, considering the phenotype of the respective parental line. It is suggested that the reversed classes of loci are involved in transgression effects visible for most of the studied traits on the phenotypic level.

Figure S1 shows that QTL may span different map distances comprising various numbers of linked loci with the same pattern of distorted segregation. There are QTL represented by 3–5 tightly linked markers, while others spread along larger map distances with higher numbers of markers showing distorted segregation. This variation of the QTL range cannot be entirely attributed to the unequal distribution of crossing-over incidents in different chromosome regions and to linkage drag. It may also result from the existence of different numbers of linked polymorphic genes that underlie QTL for individual traits. This hypothesis is based on numerous examples where overlapping QTL span somewhat different map distances (ST1.3 vs. HD1.7, PH2.1vs. SL2.2, PH4.2 vs. SL4.3 and more). On the other hand, there are coinciding QTL of exactly the same range (SL1.1–HD1.1, TKW1.7–KL1.2, PH1.3–SL1.4 and many more), which illustrates the precision of QTL detection.

The distribution of QTL on the map of rye genome is different for each trait, although, in many distinct regions, overlapping QTL for two or more traits is observed (Fig. S1, Table 3). The common feature of genetic architectures for particular traits is the presence of only a few (1–5) QTL of the D class, with much higher numbers of QTL representing R or E classes. With the exception of leaf size and chlorophyll content, the map position and a number of major QTL of class D is unique for each trait. Variation of plant height underlies one QTL of class D on chromosome 3RL and one D′ class QTL on chromosome 5RL. Stem thickness is controlled by one QTL of class D′ on chromosome 2RS and one D class QTL on chromosome 5RL. One QTL of class D controls spike length on chromosome 5RL. Chromosome 3R contains four loci of the D class for awn length. The fifth QTL of class D for this trait belongs to chromosome 7RL. The locus of class D for heading date is located on chromosome 1RS and two loci of the reversed class D′ are detected on chromosomes 5RS (1) and 7RL (1). Chromosome 1R carries the D class locus for thousand-kernel weight. Kernel length is controlled by three QTL of class D on chromosome 5RL. The presence of the D class loci for leaf size is restricted only to chromosome 1RL (3), where they coincide with one long-range QTL of class D for chlorophyll content. Another D class locus for chlorophyll content is located on chromosome 5RS. QTL of class D are distant from other loci (HD1.2.D, SL5.2.D, PH3.3.D, AL3.4.D) or they are flanked by tightly linked QTL of E (LS1.4.D, AL3.2.D) or R (KL5.5.D) classes. Independent distribution of the major loci for different quantitative traits may play a crucial role in widening the possibilities of multi-trait variation. It may form a genetic basis for generating a large variety of phenotypes with various combinations of traits values.

**Table 3:**
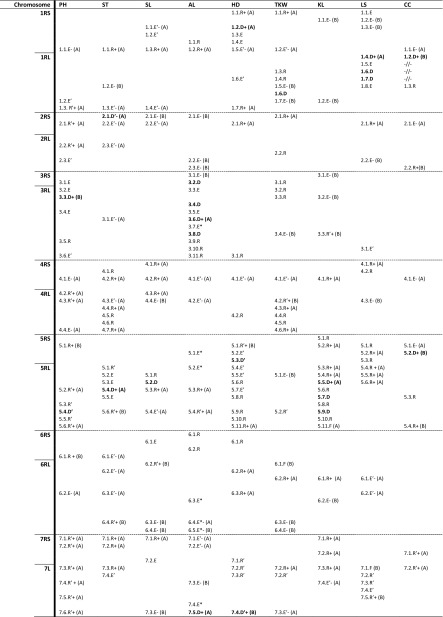
Genetic architectures of plant height (PH), stem thickness (ST), spike length (SL), awn length (AL), heading date (HD), thousand-kernel weight (TKW), kernel length (KL), leaf size (LS) and chlorophyll content (CC) in the 541 × Ot1–3 mapping population of rye. Coinciding loci have −/+ signs for negative or positive effects, respectively, of an A (line 541) or B (line Ot1–3) allele. The letters assign QTL classes

According to the genetic model presented earlier (Masojć et al. 2016), QTL of the R or E classes are interacting with the D class locus to produce positive or negative impacts on a trait, respectively. For the majority of studied traits, reversed R′ and E′ class loci were present in addition to R and E class loci (Table 3). They were especially numerous for plant height (mostly R′ loci), stem thickness, heading date and kernel length, which may be related to the apparent transgression observed for these traits in the RILs population. An additional reason for transgression might also be loci of the D′ class identified for plant height, stem thickness and heading date. Rare E* class loci, representing interaction of the E*–E* type (Masojć et al. 2016), were detected for awn length. This class is characterised by allelic segregation according to a 2:1 ratio in both interacting loci within a group of desirable phenotypes and prevalence of one allele within a group of undesirable phenotypes. QTL of hypothetical F class resulting from the D–F interaction type were identified for thousand-kernel weight (TKW6.1.F), kernel length (KL5.11.F) and leaf size (LS7.1.F), and this is the first report on that type of loci. The F class QTL are very special, for they represent interaction effects when one allele is prevailing within the lower and upper population tails. Serial distribution of at least three QTL representing the same class across a chromosome region is another interesting feature of genetic architectures for most studied traits. Series of common class QTL are found, for example, on chromosomes 7R (PH7.1–6.R′, ST7.1–3.R, HD7.1–3.R′, KL7.1–3.R), 5R (HD5.8–11.R, LS5.1–6.R) or 4R (ST4.4–7.R, TKW4.3–6.R). The mechanism of such QTL synchronisation is not known, yet it seems to be of epigenetic nature.

A large number of genomic regions with overlapping QTL for 2–8 traits were found in this study (Table 3). Such QTL are of special interest for breeders, as they may represent common genes that can ascertain improvement of more than one agronomic trait at the same time. There are several examples (1RS, 4RS, 5RL, 6RL and 7R) where the same allele at coinciding loci is acting toward positive change of 2–4 traits. Possibilities of improving at least two independent traits by selection, based on the same allele, are listed in Table 4. Genetic interactions between pairs of the studied traits presented in Table 4 probably underlie relationships found on the phenotypic level (Table 2). The main genetic mechanism of the observed traits association is possibly the close linkage between major D class loci. They are linked in case of stem thickness and spike length (5RL), plant height and awn length (3RL), and spike and kernel length (5RL).

**Table 4:** Coinciding QTL and their common effects on developmental traits in rye

Coinciding QTL	Common effects of A alleles introduced by the 541 parental line
ST1.1.R–SL1.3.R ST4.2.R–SL4.2.R ST5.4.D–SL5.3.R ST7.1.R–SL7.1.R	Increased stem thickness and increased spike length
LS1.4.D–CC1.2.D LS1.6.D–CC1.2.D LS1.7.D–CC1.2.D LS5.2.R–CC5.2.D	Increased leaf area and reduced amount of chlorophyll in leaves
SL1.3.R–AL1.2.R SL5.3.R–AL5.3.R	Increased spike length and increased awn length
PH5.2.R′–ST5.4.D PH7.1.R′–ST7.1.R PH7.2.R′–ST7.2.R PH7.3.R′–ST7.3.R	Decreased plant height and increased stem thickness
PH4.2.R′–SL4.3.R PH5.2.R′–SL5.3.R PH7.1.R′–SL7.1.R	Decreased plant height and increased spike length
PH1.3.R′–HD1.7.R PH2.1.R′–HD2.1.R PH2.2.R′–HD2.2.R PH5.6.R′–HD5.11.R	Decreased plant height and speeded up heading
TKW6.2.R–KL6.1.R TKW7.2.R–KL7.3.R	Increased thousand-kernel weight and increased kernel length

Coinciding of the D class loci may lead to situations where a given allele is positive for one trait and negative for another. Such contradictory effect of an A allele in LS1.4D and CC1.2.D loci possibly results in the light green colour of most of the plants with large leaves, whereas that of a B allele results in the much darker green colour of plants with small leaves. There are also numerous examples of coinciding loci representing different classes for each trait: SL1.1.E′–HD1.2.D, PH1.1.E–SL1.3.R, PH2.2.R′–ST2.3.E′, PH7.1.R′–ST7.1.R etc. According to the model (Masojć et al. 2016), a class of QTL depends on the specific interaction with another locus. Therefore, the same locus may be engaged in the interaction of different types within genetic networks underlying another trait. This hypothesis is supported by the observation that almost all major epistatic loci of the D class are specific for each of the studied traits. Coinciding loci of different classes can positively affect one trait and negatively another. However, the negative effect of an allele from the coinciding E class locus may be eliminated when it is recombined with a positively acting allele from the epistatic locus of class D. For instance, the unwanted effect of an A allele from the PH1.1.E locus (increased plant height) can be diminished by the B allele at the PH3.3.D locus in PH1.1AA;PH3.3BB recombinants. Then, selection for an A allele at the PH1.1/ST1.1.R/SL1.3.R locus should increase spike length and straw thickness possibly without a strong effect on plant height. Such a solution can be applied when coinciding loci are of E and R, E′ and R, E and R′, or D and E classes for different traits.

The alignment of genetic architectures for various agronomic traits and QTL classification according to the GIABDS method gives a chance to develop precise selection strategies aimed at combining several valuable traits in one genotype or population. Table 5 presents the selection strategy for developing RILs population of short rye plants with long spike, thick, strong stem, high thousand-kernel weight and early heading date. Considering that QTL and alleles are precisely identified by sets of DArT molecular markers convertible into specific SCARs, implementation of the presented strategy seems to be possible. However, the main drawback of a selection scheme based on numerous QTL is the large population size necessary for finding desirable genotypes.

**Table 5:** An example of a possible strategy for marker-assisted selection (MAS) within RILs of 541 × Ot1–3 intercross, considering QTL interaction

Objective of selection	QTL and alleles targeted by MAS in the chosen direction
Short, thick stem and long spike	PH3.3.B, PH5.4.A, PH3.5.B, PH5.1.B, PH6.1.B, ST2.1.B, ST5.4.A, SL5.2.A, (ST1.1–SL1.3)A, (ST4.2–SL4.2)A, (PH4.2–SL4.3)A, (PH7.1–ST7.1-SL7.1.)A, (PH7.2–ST7.2.)A
+ high thousand-kernel weight	TKW1.1.A, TKW1.6.A, TKW3.2.A, TKW3.3.A, TKW4.3.A, TKW4.4.A, TKW4.5.A, TKW6.2.A, TKW7.2.A
+ early heading date	HD1.2.A, HD2.1.A, HD5.3.B, HD5.8.A, HD5.9.A, HD5.10.A, HD5.11.A, HD6.1.A, HD7.4.B

A = An allele introduced by the 541 parental line; B = an allele introduced by the Ot1–3 parental line

## Discussion

This paper presents further experimental data fitting the genetic model of QTL interaction affecting alleles distribution within population tails (Masojć et al. 2016). The genetic architectures of nine quantitative traits show QTL diversification into various classes. A limited number of the D class loci and higher numbers of loci representing R and E classes control variation of quantitative traits in rye as revealed by the GIABDS analysis. According to the model, interactions between QTL of D and R classes exert positive effects while those between QTL of D and E classes exert negative effects on the trait in respect to its breeding value. Consequently, only loci of D and R classes can contribute through interaction to trait improvement and should be considered as important candidates for marker-assisted selection. Sequences of DArT markers representing these QTL constitute a good starting point for developing specific markers useful in practical breeding and for further search of functional genes.

A high-density map of DArT markers (Milczarski et al. 2011) appeared to be useful in detecting QTL defined by series of linked markers with distorted segregation within population tails. This way of QTL mapping allows for precise alignment of genetic architectures for the studied traits. Comparative analysis of genetic architectures by means of the GIABDS method reveals the unique distribution of the D class QTL for each trait, with the exception of leaf area and chlorophyll content having D class loci in the same position on chromosome 1RL. It is interesting that, for some traits, a series of the D class loci are localised on one chromosome, like for awn length, leaf area and kernel length, similarly as for pre-harvest sprouting resistance (Masojć et al. 2009). Another common feature of genetic architectures for pre-harvest sprouting, alpha-amylase activity and developmental traits is the synchronisation of QTL classes across specific regions or even entire chromosomes (Masojć et al. 2009, 2011 and this study). At this stage, it can only be speculated that the newly noticed phenomenon might reflect the involvement of epigenetic mechanisms in controlling quantitative traits variation.

Distributions of the R and E QTL classes across rye chromosomes is trait-specific, although some coinciding QTL might represent common genes with pleiotropic effects for different traits, thus being a valuable target of selection. The existence of pleiotropic genes is more probable when coinciding QTL of narrow range control more than two traits, since the probability of functional polymorphisms of multiple tightly linked genes for each trait within one bi-parental population is low. Such QTL were found on chromosomes 1RS, 1RL, 6RS, 6RL and 7RL. They represent similar or different classes, but this may result from trait-specific interactions. Overlapping QTL having wider ranges, which are more abundant in this study, probably represent groups of linked genes affecting different traits. Coinciding loci with similar allele effect probably contribute to the necessary coordination of developmental traits like in the cases of spike length and stem thickness, spike length and awn length, or spike length and kernel length. They also prove the accuracy of QTL identification by the GIABDS method, since analysis for each trait is based on an independent set of RILs representing extreme phenotypic values. Although the genetic architectures of the studied traits are partially overlapping, their vast parts are independent, which opens the possibility of generating various phenotypes through genetic recombination. It is a very promising conclusion for practical breeding, as it implies that the selection strategy proposed in Table 5 can be realised only if an RILs population of sufficient size is ascertained.

Considering the map positions of the D class loci for pre-harvest sprouting (PHS) revealed in the 541 × Ot1–3 mapping population (Masojć et al. 2009), it might be expected that selection for resistance to PHS would affect plant height (3RL), spike and kernel length (5RL), thousand-kernel weight (1RL), awn length (3RL) and leaf area (1RL) due to coinciding or linkage between QTL. However, complex genetic architectures and differences between PHS and other trait loci suggest that finding recombinants with sprouting resistance and satisfactory yielding level should be possible.

The genetic architectures of quantitative traits reported for different crosses used as mapping populations are often only partially overlapping, as shown for PHS (Myśków et al. 2012; Masojć and Milczarski 2009). This is due to the specific distribution of functional polymorphisms in genetically different plant materials. Also, a variety of marker systems used for mapping projects makes it difficult to identify coinciding QTL by comparing different maps. Nevertheless, the detection of QTL for a given trait in a similar chromosome region in different experiments is always valuable. A specific region of rye genome where two dwarfing genes *ct2* and *Ddw1* and respective QTL for plant height were reported in the distal part of chromosome 5RL (Plaschke et al. 1993; Börner et al. 1999; Tenhola-Roininen and Tanhuanpää 2010; Miedaner et al. 2012). A series of four QTL found in this region by means of the GIABDS method constitute additional proof for its significance in the genetic control of plant height. QTL PH5.5.R′ or PH5.4.D′ and PH5.6.R′ have similar map positions as *ct2* and *Ddw1* genes, respectively, although the allelic set at these QTL is not leading to dwarfism in parental line Ot1–3. The SL5.2D locus from the proximal part of chromosome 5RL is another example of similar QTL location found in two independent investigations (Börner et al. 1999 and this study). There are more cases of similar QTL position detected here and in other studies, i.e. heading date on chromosomes 1R and 6R (Myśków and Stojałowski 2016; Święcka et al. 2014), thousand-kernel weight on chromosomes 1RS, 1RL, 3RS, 5RS, 6RS and 7RL (Miedaner et al. 2012; Myśków et al. 2014), plant height on chromosomes 1RS, 2RS, 2RL, 3RL, 5RS and 7RL (Myśków et al. 2014; Miedaner et al. 2012), spike length on chromosomes 2RS, 4R, 5RL, 6R (Myśków et al. 2014) and chlorophyll content on chromosomes 1D and 5D in wheat (Osipova et al. 2016).

A common feature of genetic architectures reported to date for developmental traits in cereals is the high number of QTL distributed on the majority or all chromosomes. Kernel length in rice is controlled by 17 QTL (Zhao et al. 2017) and each chromosome group of wheat and barley contains at least one QTL for thousand-kernel weight (Mohler et al. 2016; Ogrodowicz et al. 2017; Lex et al. 2014). Numerous QTL spread across wheat, barley and rye genomes control plant height, spike length and heading date (Cui et al. 2011; Mikołajczak et al. 2017; Ogrodowicz et al. 2017; Miedaner et al. 2012; Myśków et al. 2014 and this paper). A growing amount of experimental data show that QTL forming genetic architectures of complex traits control phenotypic variation through two-loci interactions (Xing et al. 2002; Wang et al. 2010; Masojć et al. 2009, 2011, 2016). The results presented here support this conclusion by showing that the majority of detected QTL for nine traits in rye represent R and E classes, which, according to the genetic model (Masojć et al. 2016), are involved in interactions with less numerous QTL of the D class.

Comparative analysis of genetic architectures for different agronomic traits within a highly differentiated bi-parental population, carried out using the GIABDS method and a high-density map, seems to be very promising in both scientific and practical aspects. It gives the possibility of accumulating comparable data on complex functional genomics of plants traits and generates valuable results leading to the development of marker-assisted selection strategies aimed at parallel improvement of several traits in one breeding programme. Such programmes should result in pyramiding positively acting genes and their interactions in one variety.

## Electronic supplementary material

Below is the link to the electronic supplementary material.

Fig. S1 Alignment of genetic architectures of plant height (PH), stem thickness (ST), spike length (SL), awn length (AL), heading date (HD), thousand-kernel weight (TKW), kernel length (KL), leaf size (LS) and chlorophyll content (CC) on a high-density DArT-based genetic map of rye developed using the 541 × Ot1–3 population of RILs. The vertical lines assign a block of linked molecular markers, for which allelic segregation in population tails is significantly distorted from the 1:1 segregation ratio, to QTL marked by the trait symbol, chromosome number, consecutive identification number and a class symbol according to the GIABDS method. (* = QTL classes showing a 2:1 segregation ratio in the population tail; ′ = reversed allele effect (XLSX 68 kb)
